# The Dual Role of Human UDP-Glucuronosyltransferase-Mediated Metabolism in Tumor Drug Resistance: Mechanisms and Prospects

**DOI:** 10.3390/ijms27156955

**Published:** 2026-08-03

**Authors:** Zhike Wang, Jin Zhong, Chenran Ren, Hao Shi, Xiong Fang, Xiao Xiao, Xia Liu, Deliang Cao, Xi Zeng

**Affiliations:** 1 Hunan Province Key Laboratory of Tumor Cellular & Molecular Pathology, Cancer Research Institute, Hengyang Medical School, University of South China, Hengyang 421001, China; 2 Department of Oncology, The First Affiliated Hospital, Hengyang Medical School, University of South China, Hengyang 421001, China

**Keywords:** UDP-glucuronosyltransferase (UGT), tumor drug resistance, glucuronidation, intervention strategies, drug transporters, transcription factors, chemotherapy drugs

## Abstract

UDP-glucuronosyltransferases (UGTs) are a key family of phase II metabolic enzymes in humans that play a central role in maintenance of metabolic homeostasis and drug disposition by catalyzing glucuronidation of endogenous and exogenous substances. UGT-mediated metabolism of antitumor drugs plays a dual role in tumor drug resistance, emerging as a hotspot in cancer therapy. This review outlines the structural characteristics, classification system, tissue distribution, and multilevel regulation of UGTs, with a focus on their bidirectional roles in tumor drug resistance, i.e., promotion of resistance through metabolic clearance and inhibition of resistance through metabolic activation. This review also discusses strategies of multidimensional tumor resistance intervention based on UGTs, and we also discussed the challenges in the clinical translation of current UGT-targeting strategies. To date, most data on UGTs were derived from in vitro and preclinical models; clinical validation remains limited, and the dual roles of UGTs are highly context-dependent. This review article provides a perspective for comprehensive understanding of UGT-mediated tumor resistance and offers theoretical foundations and practical directions for development of novel antitumor strategies.

## 1. Introduction

Chemotherapy is widely used to treat various types of cancers, but its efficacy is often limited due to drug resistance [1]. Tumor drug resistance can be categorized into primary and acquired resistance [2]. Primary resistance, also named intrinsic drug resistance, refers to the insensitivity of tumor cells to chemotherapy drugs at the onset of treatment, whereas acquired resistance denotes the gradual development of resistance by tumor cells during the treatment [3]. The mechanisms of drug resistance are complex and multifaceted, including the overexpression of drug efflux pumps [4], altered activity of drug-metabolizing enzymes [5], enhanced DNA repair mechanisms [6], and abnormal apoptosis pathways [7]. These mechanisms are intertwined and synergistic, enabling tumor cells to evade the cytotoxic effects of chemotherapy drugs [8].

UDP-glucuronosyltransferases (UGTs) are a crucial family of drug metabolic enzymes [9]. UGTs catalyze the conjugation of endogenous and exogenous substrates with glucuronic acid, enhancing their water solubility to facilitate excretion and reduce their toxicity. Beyond drug metabolism, UGTs also play essential physiological roles in the glucuronidation of steroids, bile acids, and neurotransmitters, thereby regulating hormonal balance, bile acid excretion, and neuronal signaling. Therefore, UGT has emerged as a focal point in tumor drug resistance, and increasing evidence indicates that UGT-mediated metabolism plays a critical role in tumor resistance. In addition to metabolism of chemotherapeutic drugs, UGTs also influence drug sensitivity of tumor cells by regulation of intracellular signaling pathways [10]. Therefore, an in-depth insight into the mechanisms underlying UGT-mediated tumor resistance holds significant theoretical and practical importance in development of novel therapeutic strategies. By targeting UGTs or associated metabolic pathways, it is possible to reverse tumor cell resistance, enhance the efficacy of chemotherapy, and offer new hope to patients with cancer. However, UGTs play a dual role in tumor drug resistance, and the specific molecular mechanisms underlying the dual role of UGT-mediated tumor resistance remain unclear. The differentials of the action of UGTs across different tumor types and various chemotherapeutic agents need to be elucidated; the interactions between UGTs and other drug resistance-associated proteins or signaling pathways require a comprehensive understanding; the efficacy and safety of UGT-targeted therapeutic strategies in clinical applications necessitate more clinical trials to advance the translation from bench to beds.

This review aims to systematically address the dual roles of human UDP-glucuronosyltransferase-mediated metabolic regulation in tumor drug resistance and delve into the underlying mechanisms based on the research progress in this field. This study seeks to provide new insights and theoretical foundations for the study and treatment of drug resistance in tumors.

## 2. Overview of UDP-Glucuronosyltransferases

### 2.1. Structure and Function of UDP-Glucuronosyltransferases

**Figure 1:**
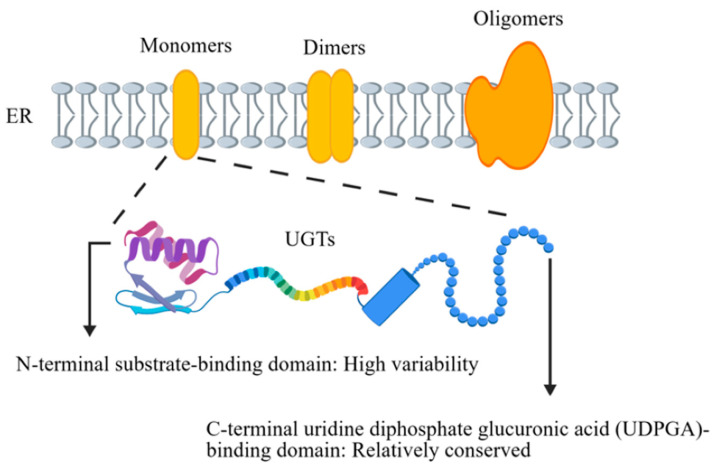
Structure of UDP-glucuronosyltransferases. (Created with BioGDP.com) Dimers are functional UGT complexes formed by two monomers, essential for glucuronidation activity. Oligomers are higher-order UGT assemblies (≥3 subunits) that modulate substrate specificity and drug metabolism.

UDP-glucuronosyltransferases (UGTs) are phase II metabolic enzymes in the human body that belong to a class of glycosyltransferases. They primarily exist in the endoplasmic reticulum as monomers [11], dimers, or oligomers, maybe forming homodimers and heterodimers [12,13]. The UGT family comprises numerous members with amino acid sequence homology but distinct substrate specificity and catalytic activity. The UGT protein structure comprises an N-terminal substrate-binding domain and a C-terminal uridine diphosphate glucuronic acid (UDPGA)-binding domain. The N-terminal region exhibits high variability, which determines the substrate specificity of different UGT isoforms [14]. The C-terminal region is relatively conserved and is responsible for binding of UDPGA which supplies the glucuronic acid moiety for glucuronic acid conjugation (Figure 1) [15].

The primary function of UGTs is to catalyze glucuronidation, wherein endogenous or exogenous substances (substrates) are conjugated with the glucuronic acid moiety of UDPGA to form glucuronide conjugates. This glucuronidation enhances water solubility of the substrate, facilitating excretion via urine or bile [16]. Consequently, UGTs play a critical role in maintenance of dynamic equilibrium of endogenous compounds, such as bilirubin and bile acids, and in the disposal of exogenous compounds, such as drugs and carcinogens [17,18]. For instance, bilirubin undergoes glucuronidation in the liver via UGT1A1-catalyzed conjugation with glucuronic acid to form water-soluble bilirubin glucuronide for excretion. Mutations in the UGT1A1 gene that reduce or eliminate enzyme activity prevent effective bilirubin glucuronidation, leading to bilirubin accumulation and hyperbilirubinemia [19]. Many drugs, such as acetaminophen and irinotecan [20], require UGT-catalyzed glucuronidation in their metabolism. A portion of acetaminophen is metabolized in vivo by UGT-catalyzed glucuronidation to form glucuronide conjugates for excretion. Abnormalities in the glucuronidation lead to the accumulation of toxic acetaminophen metabolites and liver damage [21].

### 2.2. Classification and Tissue Distribution of UDP-Glucuronosyltransferases

Based on amino acid sequence homology, UGTs are classified into four families (UGT1, UGT2, UGT3, and UGT8) and five subfamilies (1A, 2A, 2B, 3A, and 8A). The nine protein-coding genes of the UGT1A family (1A1, 1A3–1A10) are generated by splicing of the unique exon 1 to shared other exons (exons 2–5). The UGT2B family comprises seven functional protein-coding genes (2B4, 2B7, 2B10, 2B11, 2B15, 2B17, and 2B28), each of which is encoded by six distinct exons [22]. UGT1 primarily mediates the metabolism of phenolic compounds [23] and bilirubin [24], whereas UGT2 is primarily involved in steroid metabolism [25]. Two members of the UGT3 family [26], UGT3A1 and UGT3A2, utilize UDP-N-acetylglucosamine [27] and UDP-glucose/xylose to conjugate bile acids, steroids, and bioflavonoids [28]. The UGT8 family member UGT8A1 uses UDP-galactose to synthesize galactosylceramides and bile acids (Table 1) [29].

UGT is widely distributed throughout various tissues and organs in the human body, with the liver, gastrointestinal tract, and kidneys as the primary organs that highly express UGTs [30]. Adult liver tissues contain multiple UGTs, including 1A1, 1A6, and 1A9 from the UGT1A family and 2B4, 2B7, and 2B15 from the UGT2B family. 1A3, 1A6, 2B10, 2B11, and 2B17 can also be detected in the liver at a relatively low expression level [31]. UGT2B4 is the most abundant subtype in the liver, accounting for approximately 40% of the total, followed by UGT2B7, UGT2B17, UGT1A1, and then UGT1A9. In the intestine, UGT1A1, UGT1A10, UGT2B7, UGT2B17, and UGT1A3 and UGT1A4 at a low level are expressed, but only three UGTs, i.e., UGT1A9, UGT2B7, and UGT1A6, are detected in the kidney [32]. The differential distribution of UGTs across tissues defines their specificity and importance in substrate metabolism within different organs [33].

### 2.3. Regulation of UGT Expression and Activity

**Figure 2:**
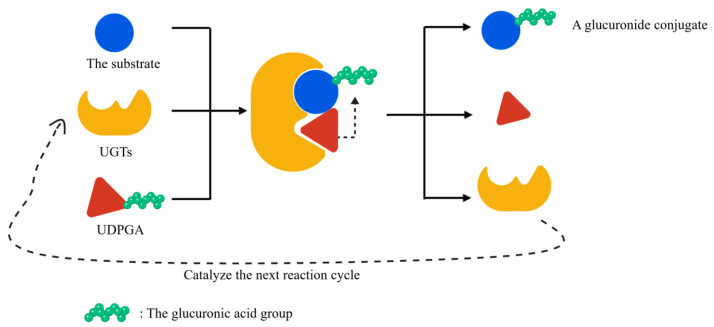
UGT-catalyzed metabolism. (Created with BioGDP.com) The substrate (blue circle) indicates an endogenous or exogenous compound (e.g., drugs, bilirubins) that undergoes glucuronidation. UDP-glucuronic acid (UDPGA) denotes the donor molecule that provides the glucuronic acid group shown as the structure containing UDP and a glucuronic acid group (the green chain). UGTs (yellow shape) are a UDP-glucuronosyltransferase enzyme located in the endoplasmic reticulum that catalyzes the transfer reaction. A glucuronide conjugate (blue circle and green chain) indicates the product of the reaction, which is more water-soluble. After release of the glucuronide conjugate, the UGT enzyme is free to enter another circle of reaction. UGTs, UDP-glucuronosyltransferases and UDPGA, UDP-glucuronic acid.

UGT-mediated metabolic regulation is a complex process primarily achieved through two mechanisms: catalytic metabolism and gene expression regulation. The UGT-catalyzed metabolism includes the substrate binding to the N-terminal substrate-binding domain, UDP-glucuronic acid (UDPGA) binding to the C-terminal UDPGA-binding domain, transfer of glucuronic acid group from UDPGA to the substrate molecule, forming a glucuronide conjugate, and then dissociation of glucuronide conjugate from the UGT [34]. This process is influenced by multiple factors, including substrates and UDPGA concentrations, enzyme activity, and reaction conditions, such as pH and temperature. Excessively high substrate concentrations may induce substrate inhibition, thereby reducing the rate of glucuronic acid conjugation. Conversely, insufficient UDPGA concentrations limit the progress of the reaction (Figure 2) [35].

Gene expression regulation is crucial for the metabolic activity of UGTs. The expression of UGT genes is regulated at multiple levels, including pre-transcriptional, transcriptional, post-transcriptional, and post-translational levels. At the pre-transcriptional level, epigenetic modifications, such as DNA methylation and histone modifications, can influence UGT gene expression [36]. DNA methylation typically suppresses gene transcription, and hypermethylation of the UGT gene promoter region reduces UGT gene expression [37]. At the transcriptional level, tissue-specific factors and nuclear receptors regulate UGT gene expression. Tissue-specific factors, such as hepatic nuclear factor (HNF), bind to UGT gene promoters or enhancers to promote transcription [38]. Nuclear receptors, such as the pregnane X receptor (PXR) and constitutive androgen receptor (CAR) [39], also regulate UGT transcription by binding to their respective ligands. At the post-transcriptional level, non-coding RNAs, such as microRNAs, regulate UGT expression by either inhibition of mRNA translation through complementary pair with UGT mRNAs or promotion of mRNA degradation [40]. At the post-translational level, modifications, such as glycosylation and phosphorylation, can alter the stability, activity, and localization of UGT proteins, thereby influencing their metabolic function [41]. These multilevel regulatory mechanisms coordinate to maintain the equilibrium of UGT-mediated metabolic control within the body.

## 3. Mechanisms of Tumor Drug Resistance

### 3.1. Drug Efflux and UGT-Mediated Glucuronidation

Drug efflux by ATP-binding cassette (ABC) transporters is a key mechanism of multidrug resistance and is also related to the UGT-mediated glucuronidation. Glucuronide metabolites generated by UGTs are highly polar and negatively charged, preventing passive diffusion across cell membranes. The efflux of glucuronide conjugates needs the active efflux by specific ABC transporters [42]. The major ABC transporters involved in the efflux of UGT-generated glucuronides include P-glycoprotein (P-gp, also known as ABCB1), a protein primarily known as a transporter of hydrophobic drug [43], and multidrug resistance-associated proteins (i.e., MRP1, ABCC1; MRP2, ABCC2; MRP3, ABCC3; MRP4, ABCC4). MRP1 and MRP3 export glucuronidated drugs, such as SN-38 glucuronide, while MRP2 (also called cMOAT, canalicular multispecific organic anion transporter) is responsible for excretion of biliary glucuronides [44]. Breast cancer resistance protein (BCRP, also known as ABCG2) also transports a variety of glucuronidated substrates, including the glucuronides chemotherapeutic agents [45]. Hence, drug efflux is a critical drug-resistant mechanism, pumping out chemotherapeutic agents and their glucuronides produced by UTGs.

### 3.2. Drug Metabolism

Drug-metabolizing enzymes play a crucial role in the development of tumor drug resistance, with cytochrome P450 enzymes (CYP) and UDP-glucuronosyltransferases (UGTs) being key members [46]. CYP is a group of heme-containing enzymes that convert drugs into more hydrophilic metabolites that are easier to excrete, thereby reducing drug bioactivity. CYP3A4 metabolizes various chemotherapeutic agents, such as cyclosporine and tacrolimus [47]. In hepatocellular carcinoma cells, elevated CYP3A4 expression accelerates the metabolism of these drugs, inducing drug resistance [48].

UGTs are important drug metabolic enzymes, reducing the drug exposure to tumor cells. When UGT expression or activity is upregulated in tumor cells, the retention time and effective concentrations of drugs within the cells are reduced.

### 3.3. Target Mutations, Signaling Abnormalities, and Links to UGT Activity

Target mutations are a major molecular mechanism driving tumor drug resistance. Genetic alterations in drug action targets can alter protein structure and function, thus impairing therapeutic efficacy. In non-small cell lung cancer (NSCLC), the epidermal growth factor receptor (EGFR) is a therapeutic target [49]. The T790M mutation of EGFR close to the ATP-binding site within the kinase domain increases the kinase affinity for adenosine triphosphate (ATP) but decreases the binding capacity to inhibitors, resulting in tumor cell resistance to gefitinib and erlotinib [50]. These mutations do not happen in UGTs but often co-occur with altered UGT expression in resistant tumors. Targeting both pathways may offer synergistic effects.

Abnormal activation of signaling pathways also plays a crucial role in drug resistance. Tumor cells can evade the effects of drugs by activating alternative signaling pathways or reactivating the targeted pathways. In breast cancer, the abnormal activation of the PI3K/Akt/mTOR signaling pathway is closely associated with trastuzumab resistance. Activation of this pathway promotes tumor cell proliferation, survival, and anti-apoptotic capacity, thereby reducing sensitivity to trastuzumab [51]. Activation of PI3K phosphorylates downstream Akt, which in turn activates mTOR to promote cellular growth, metabolism and survival. When the PI3K/Akt/mTOR signaling pathway is excessively activated in tumor cells, even if trastuzumab blocks the HER2 signaling pathway, tumor cells can still maintain growth and survival through this alternative signaling pathway, leading to resistance [52]. Recent evidence indicates that PI3K/Akt signaling can transcriptionally regulate UGT isoforms (e.g., UGT1A1 and UGT2B7), creating a crosstalk with UGT-mediated drug metabolism.

## 4. Dual Role of UGTs in Tumor Drug Resistance

### 4.1. Tumor Drug Resistance Promoted by UGTs

#### 4.1.1. Metabolism of Chemotherapy Drugs Catalyzed by UGTs

A primary mechanism through which UGTs promote tumor drug resistance is catalytic inactivation of chemotherapeutic agents. UGTs catalyze the glucuronidation of chemical drugs, and thus low expression and reduced activity of UGT enzymes in tumor tissues may enhance the antitumor efficacy of drugs. In breast cancer, UGT expression may be up to five times lower than that in adjacent non-cancerous tissues, and thus metabolic inactivation of drug by glucuronidation is diminished [53]. In hepatocellular carcinoma and renal cell carcinoma, the low expression of UGT2B7 and UGT1A9 also diminishes the local metabolic clearance of chemotherapeutic agents and elevates local drug concentrations and activities [54].

Irinotecan (CPT-11) is a semi-synthetic camptothecin derivative that serves as a prodrug for topoisomerase I (Topo I) inhibition. It is currently a first-line chemotherapy drug for colorectal, lung and gastric cancers [55]. Its active metabolite, 7-ethyl-10-hydroxycamptothecin (SN-38), undergoes glucuronidation catalyzed by UGT1A1, UGT1A7, and UGT1A9, forming the inactive SN-38 glucuronide. In the colon cancer cell line HT-29 and the drug-resistant lung cancer cell line PC-7/CPT [56], high expression of UGT1A1, UGT1A9, and UGT1A10 significantly accelerates the metabolic inactivation of SN-38, leading to cellular resistance. Studies have also indicated that irinotecan metabolism may involve synergistic action of other UGT isoenzymes, aggravating the drug metabolic fate within tumor cells [57].

Belinostat is a pan-histone deacetylase (HDAC) inhibitor used for relapsed or refractory peripheral T-cell lymphoma (PTCL) [58]. Primarily catalyzed by UGT1A1, hydroxymethylglucuronidation generates the corresponding glucuronide metabolites, leading to the rapid clearance of the parent drug. In tumor tissues, high UGT1A1 expression accelerates belinostat metabolism, reducing its antitumor efficacy [58,59]. Moreover, UGT1A1 expression levels are positively correlated with the metabolic rate of belinostat within tumor cells. With UGT1A1 overexpression, tumors exhibit more pronounced resistance to belinostat [60].

Etoposide is a topoisomerase II (Topo II) inhibitor that blocks DNA replication and transcription by forming a ternary complex with Topo II and DNA, inducing apoptosis of tumor cells. This drug is widely used in the clinical treatment of malignant tumors, such as small cell lung cancer, non-small cell lung cancer, lymphoma, and ovarian cancer [61]. Etoposide glucuronidation is primarily catalyzed by UGT1A1. UGT1A3 and UGT1A8 also contribute, but to a lesser extent [62,63]. Glucuronidated etoposide is converted into an inactive glucuronide for excretion, diminishing inhibitory effect on topoisomerase II and antitumor efficacy [63].

Epirubicin (epirubicin hydrochloride) is an anthracycline-based broad-spectrum antineoplastic agent that targets topoisomerase IIα (Topo IIα) by stabilizing the Topo IIα-DNA double-strand break complex and blocks DNA replication and transcription processes, inducing tumor cell apoptosis. This drug is widely used in the clinical treatment of solid tumors, such as breast, lung, ovarian, and gastric cancers. However, its efficacy is limited by tumor cell resistance [64]. In the liver, epirubicin is extensively metabolized by UGT2B7 into glucuronide conjugates and excreted. If UGT2B7 expression is upregulated in tumor tissues, the metabolic clearance of epirubicin is accelerated and tumor resistance is induced. Co-expression of UGT2B7 with resistance-related proteins synergistically promote tumor resistance [65].

In summary, metabolic inactivation of antitumor agents by UGTs is critical in drug resistance, but most of the findings come from human cancer cell lines, and UGT1A1 is the unique UGT enzyme with clinical pharmacokinetic evidence. Extensive preclinical and clinical studies are warranted to draw a solid conclusion.

#### 4.1.2. Efflux of Glucuronide Metabolites by Drug Transporters

β-glucuronidase in cells hydrolyzes glucuronidated drugs to restore their pharmacological activity, and high glucuronide hydrolysis enhances the retention of active drugs [53]. Therefore, UGT-induced drug resistance is often coupled with multidrug resistance (MDR) networks. Glucuronide metabolites produced by UGTs are highly polar and negatively charged, preventing passive diffusion across cell membranes. Their efflux requires ABC transporters (e.g., MRP family and hepatic cMOAT), so-called a “metabolism-efflux” mechanism for anticancer drug efflux. For example, in the colon cancer cell line HT29 with high UGT expression, the active metabolite SN-38 of irinotecan forms glucuronide conjugates by UGTs and is then exported by transporters MRP1 and MRP3. In contrast, HCT116 cells with low UGT expression exhibited minimal SN-38 glucuronidation but substantial intracellular accumulation of the parent drug [66].

NU/ICRF 505 is an anthraquinone–tyrosine conjugate topoisomerase I (Topo I) inhibitor, and its antitumor mechanism resembles the active metabolite SN-38 of irinotecan, inducing DNA damage and apoptosis by stabilizing the topoisomerase I-DNA cleavage complex [67]. Due to the high hydrophobicity, this compound cannot be directly recognized by ABC transporters and has to undergo UGT-catalyzed glucuronidation to generate a polar metabolite for efflux by MRP1 and MRP3 [68]. Therefore, glucuronidation is an essential step for NU/ICRF 505 clearance from cells, and the interaction between UGT and transporters is the key mechanism underlying the intrinsic resistance of tumor cells to this drug.

Emodin, a natural anthraquinone compound that is widely present in plants, such as rhubarb and Polygonum cuspidatum, exhibits multiple biological activities, including antitumor, anti-inflammatory, and antioxidant effects. It serves as a classic model molecule for studies on plant-derived anticancer agents [69]. The primary detoxification pathway for emodin relies on UGT-catalyzed glucuronidation coupled with MRP transporters (MRP2 and MRP3/4). UGTs extensively catalyze the glucuronidation of emodin in the gut and liver. The resultant conjugates are exported via MRP2 and MRP3/4. This efflux of emodin conjugates from the gut circulation results in extremely low oral bioavailability. In Caco-2 cells, emodin primarily enters cells via passive diffusion, whereas its glucuronide conjugate is effluxed by MRP2 and MRP3/4, diminishing its antitumor activity [70].

#### 4.1.3. Induction of UGTs by Chemotherapy Drugs

UGTs may be induced by therapeutic drugs. Chemotherapy drugs can activate a series of transcription factors, such as the aryl hydrocarbon receptor (AhR), pregnane X receptor (PXR), tumor suppressor protein p53, and glioma-associated oncogene 1 (GLI1) [71]. These transcription factors in turn induce UGT expression. Antineoplastic agent methotrexate is a classic folate antagonist that inhibits de novo purine and pyrimidine synthesis by blocking dihydrofolate reductase (DHFR), thereby suppressing DNA replication and cell proliferation. It is widely used in the treatment of malignant tumors, such as leukemia, lymphoma, and breast cancer, as well as autoimmune diseases. Methotrexate can activate the AhR signaling pathway and then induce the expression of UGT1A6, which catalyzes the glucuronidation inactivation of methotrexate and promotes acquired drug resistance [72]. The AhR pathway may also be activated by oxidative stress induced by drugs, further inducing UGT1A6 expression and function [73].

GLI1 is a core transcription factor in the Hedgehog (Hh) signaling pathway and plays a crucial role in embryonic development and tumor progression [74]. In acute myeloid leukemia (AML), GLI1 contributes to drug resistance through two distinct mechanisms, i.e., transcriptional activation of canonical Hh target genes and transcription-independent protein stabilization. GLI1 acts as a key transcriptional activator that drives the transcription of numerous target genes of the canonical Hedgehog signaling, mediating drug sensitivity of AML cells. GLI1 does not transactivate UGT1A genes but upregulates the expression of calreticulin (CALR), a molecular chaperone. CALR then binds to and stabilizes UGT1A proteins by inhibition of ubiquitin-dependent degradation, leading to increased UGT1A protein and enhanced glucuronidation activity. This mechanism contributes to resistance to ribavirin and cytarabine [75,76].

The irinotecan metabolite SN-38 activates the nuclear receptor PXR, thereby promoting the transcriptional expression of UGT1A1, UGT1A7, and ABC transporter family members, MDR1 and MRP2. The synergistic upregulation of UGTs and transporters accelerates irinotecan metabolism and efflux, leading to acquired resistance of tumor cells. Furthermore, the activation of the PXR pathway can influence the expression of other metabolism-related genes within tumor cells, thereby altering the overall metabolic profile and promoting resistance [77].

Epirubicin activates p53, upregulating UGT2B7, which in turn catalyzes the glucuronidation metabolism and efflux of epirubicin, inducing acquired resistance to epirubicin in tumor cells [78]. The regulation of UGT2B7 by p53 may involve non-coding RNA, forming a complex regulatory network to tune up UGT2B7 expression (Table 2) [79].

### 4.2. Metabolic Activation by UGTs: A Rare and Weakly Supported Counterbalance

UGTs promote drug resistance through metabolic inactivation. In contrast, UGTs may metabolically produce metabolites with equal or even greater antitumor activity than the parental compound.

#### 4.2.1. Tamoxifen: A Mechanistically Plausible but Context-Dependent Example

Tamoxifen is a selective estrogen receptor modulator (SERM) and a first-line drug for endocrine therapy in estrogen receptor (ER)-positive breast cancer. The action of tamoxifen involves competitive binding to ER and blocks estrogen-mediated cell proliferation signaling pathways, inducing tumor cell cycle arrest and apoptosis [80]. Tamoxifen N-glucuronidation is primarily catalyzed by UGT1A4, UGT1A8, UGT1A10, and UGT2B7 [81,82] (Table 3). The abundance of N-glucuronide versus other metabolites (e.g., 4-hydroxytamoxifen and its glucuronides) varies substantially across individuals due to UGT1A4 genetic polymorphisms and tissue-specific expression. The N-glucuronide exhibits a 15-fold higher affinity to estrogen receptors than O-glucuronide, generating enhanced antibreast cancer activity [83]. However, it is currently unknown whether N-glucuronide reaches sufficient concentrations in tumor cells to exert meaningful ER blockade. Pharmacogenetic studies have associated UGT1A4 variants with tamoxifen treatment outcomes. Prospective clinical trial is currently lacking.

#### 4.2.2. Acridone Derivatives: Preliminary Observations

Acridone drugs are a class of heterocyclic compounds centered on the acridone core, including natural plant extracts (e.g., bergapten from Rutaceae plants) and synthetic derivatives (e.g., 9-aminoacridone and methoxyacridone). These compounds have been identified as promising antitumor agents in preclinical studies and clinical trials [84]. Their primary antitumor mechanism involves intercalation between DNA double-stranded base pairs, thereby disrupting DNA replication and transcription. They also target topoisomerase I/II (Topo I/II), stabilizing Topo–DNA fracture complexes to induce DNA damage and apoptosis of tumor cells. These compounds exhibit impressive activity in the treatment of solid tumors, such as lung, colon, and liver cancers [85]. The primary catalytic isoenzyme is UGT1A10, and its glucuronide derivative of triazoloacridone (C-1305) exhibits higher antitumor activity than the natural compound [86]. This suggests that specific UGT isoenzymes may enhance drug sensitivity by generating more potent metabolites. The underlying mechanisms may stem from the altered affinity of metabolites to specific targets, enabling more effective inhibition of tumor cell growth and survival (Table 3).

**Table 3:** UGTs-mediated inhibition of tumor drug resistance.

Drugs	UGT Isoforms	Core Effects	Refs.
Tamoxifen	UGT1A4, UGT1A8, UGT1A10, UGT2B7	Tamoxifen N-glucuronidation is primarily catalyzed by UGT1A4, UGT1A8, UGT1A10, and UGT2B7. The N-glucuronide exhibits a 15-fold higher affinity for estrogen receptors than O-glucuronide.	[80,81,82,83]
Acridone drugs	UGT1A10	The primary catalytic isoenzyme is UGT1A10, and its glucuronide derivative of triazoloacridone (C-1305) exhibits higher antitumor activity than the natural compound.	[86]

#### 4.2.3. A Summary

UGTs play a dual role in the drug sensitivity of tumors, which is UGT isoform- and tissue-context dependent. UGTs as a mediator of drug resistance is more widely evident, whilst UGTs as a promotor of antitumor activity of drugs seems more preliminary. Nevertheless, the concept of dual role of UGTs is intellectually appealing, and the examples discussed above represent compelling evidence of UGT-mediated sensitivity of anti-tumor agents, serving at least as a hypothesis-generating finding.

## 5. UGT-Based Strategies for Intervention of Tumor Drug Resistance

UGT-mediated glucuronidation has emerged as a core mechanism of acquired drug resistance in tumor cells. Abnormal expression of UGTs or enhanced enzyme activity accelerates the metabolic inactivation of chemotherapeutic drugs, leading to treatment failure [87,88]. Intervention strategies targeting UGTs have become a hotspot in the field of tumor drug resistance. A multidimensional intervention system has now emerged, ranging from direct enzyme inhibition to upstream pathway regulation and drug structural optimization for personalized therapy [89,90].

### 5.1. Evidence Hierarchy for UGT-Targeted Strategies

The differential evidence levels are used below in the discussion of this section. Validated approaches indicate the strategies that have demonstrated efficacy in clinical trials (phase I–III) or are approved for clinical use in the context of UGT-mediated resistance. Currently, no UGT-targeted resistance intervention meets this criterion. Preclinical findings denote the strategies supported by in vivo animal studies (e.g., xenograft models and pharmacokinetic/pharmacodynamic studies) or robust ex vivo human tissue data, which is also lacking thus far. Hypothetical or emerging strategies are the approaches based on in vitro cell-based assays, structural biology predictions, or conceptual proposals without in vivo validation. The vast majority of UGT-targeted interventions remain at the preclinical or hypothetical stages.

### 5.2. Subtype-Specific UGT Inhibitors

The development of subtype-specific UGT enzyme inhibitors represents a conceptual direction for reversing UGT-mediated drug resistance [91]. Traditional pan-UGT inhibitors (e.g., probenecid) lack subtype selectivity and may interfere with drug metabolism in normal tissues, limiting their clinical application [92]. In recent years, the integration of medicinal chemistry and structural biology has led to breakthroughs in the development of subtype-specific UGT inhibitors. For instance, through medicinal chemistry optimization guided by NMR structural biology, a series of novel UGT1A1 small-molecule inhibitors were identified. These compounds bind tightly to the active site of UGT1A1 and specifically inhibit the glucuronidation of SN-38, enhancing the cytotoxic effect of irinotecan in drug-resistant colon cancer cells [93]. Similarly, selective inhibitors targeting UGT1A4 or UGT1A9 have been proposed [94].

Hymecromone, a natural product, has been shown to downregulate UGT1A9 expression and enzyme activity in cell lines and block sorafenib glucuronidation, thus inhibiting the downstream HAS3-HA signaling pathway [95]. Again, evidence is limited in in vitro and a single xenograft study, and no clinical data are available. To date none of these compounds have progressed beyond in vitro characterization [96].

### 5.3. Targeting Upstream Regulatory Pathways

In addition to direct enzyme inhibition, targeting the upstream regulatory pathways of UGT offers a novel strategy to reverse multidrug resistance. GLI1, a core transcription factor in the Hedgehog signaling pathway, is a key regulator of UDP-glucuronosyltransferase (UGT) gene expression. In acute myeloid leukemia (AML) and colorectal cancer, GLI1 binds to the promoter regions of UGT1A1, UGT1A9, and UGT2B17 to induce their transcriptional expression. This enhances the glucuronidation metabolism of drugs, thereby mediating multidrug resistance [75]. The GLI1 inhibitor GANNT61 has been shown to downregulate UGT1A expression in AML cell lines and restore cytarabine sensitivity. Further studies have revealed that GLI1-induced glucuronidation affects multiple chemotherapeutic agents, including cytarabine and ribavirin [97]. However, these findings are exclusively from cell-based studies [76], and approaches are currently at the hypothetical-to-early-preclinical stage.

### 5.4. Structural Optimization to Avoid UGT Metabolism

Chemical modifications of drugs to reduce UGT-mediated glucuronidation represent a rationale of drug design strategies. Campuritine A-4 (CA-4), a natural antitubulin agent, is rapidly inactivated by UGT1A9. Structural modifications into a β-lactam analog can effectively avoid metabolic inactivation by UGT1A9 in drug-resistant colon cancer cells (HT-29) [98]. Again, this is a promising proof-of-concept study requiring further development.

### 5.5. Targeting Non-Classical UGT Activity

UGTs may promote resistance independently of enzymatic activity. For example, UGT2B17 is overexpressed in castration-resistant prostate cancer (CRPC) and activates the Src-ATR signaling axis via protein–protein interaction. UGT2B17 activates the Src kinase via its C-terminal domain, recruits phosphatase PTPN1 to dephosphorylate the Src inhibitory site (Tyr530), and then activates the Src-ATR signaling axis promoting DNA damage repair and resistance of anti-androgens and chemotherapeutic agents [99,100]. The NMR-based resolution of the chemical shifts in the UGT2B17 C-terminal domain provides a critical structural foundation for development of inhibitors targeting this domain, potentially expanding intervention strategies for non-classical UGT activity [101].

### 5.6. Combination Therapies and Personalized Approaches

Combination therapies that pair UGT modulators with chemotherapy, targeted therapy, or immunotherapy are conceptually attractive [102]. In colorectal cancer, co-administration of UGT1A1 inhibitors with irinotecan has been explored to reduce dose-limiting toxicities (e.g., neutropenia and diarrhea) [103]. Notably, this application is aimed to modulate hepatic UGT1A1 activity to improve irinotecan tolerability. Personalized treatment based on UGT genotypes (e.g., UGT1A1*28 for irinotecan dosing) is validated in clinical pharmacogenetics.

## 6. Conclusions and Outlook

UDP-glucuronosyltransferases (UGTs) are core human phase II metabolic enzymes and have dual effects in antitumor drug resistance. The pro-resistance function (drug inactivation via glucuronidation) is well documented across multiple drugs (e.g., irinotecan, epirubicin, etoposide) and UGT isoforms (1A1, 1A9, 2B7). The metabolic activation is limited to a few drug–UGT isoform pairs (e.g., tamoxifen with UGT1A4) but is conceptually critical to UGT-targeted manipulation of drug resistance.

Clinically, UGT genotypes (e.g., UGT1A1*28) are used to guide irinotecan dosage for toxicity management. However, most UGT-targeted intervention strategies remain at the preclinical or hypothetical stage. The future efforts may focus on the clinical translation of achieved UGT-targeted strategies.

Looking ahead, technological development in drug design may drive breakthroughs in UGT-based tumor resistance modulations. The integration of structural biology with AI-assisted drug design holds a promise for the development of UGT inhibitors with higher specificity and affinity. Single-cell multi-omics technologies may further unravel the expression patterns and functional differences among UGT subtypes in response to tumor heterogeneity. Furthermore, an integration of UGT intervention strategies with novel therapies (e.g., proton radiotherapy and CAR-T cell therapy) holds a promise for advance of UGT-guided precision medicine in cancer therapy.

## Figures and Tables

**Table 1:** Classification and substrate distribution of UDP-glucuronosyltransferases.

UGTs	Representative Isoforms	Substrate Distribution and Metabolic Functions	References
UGT1	UGT1A1, UGT1A3, UGT1A4, UGT1A5, UGT1A6, UGT1A7, UGT1A8, UGT1A9, UGT1A10	Metabolism of phenolic compounds and bilirubin	[23,24]
UGT2	UGT2B4, UGT2B7, UGT2B10, GT2B11, UGT2B15, GT2B17, UGT2B28	Steroid metabolism	[25]
UGT3	UGT3A1, UGT3A2	Metabolism of bile acids, steroids, and bioflavonoids	[26,27,28]
UGT8	UGT8A1	Synthesis of galactosylceramides and bile acids	[29]

**Table 2:** UGTs-mediated promotion of tumor drug resistance.

Relevant Drugs	UGT Isoforms	Core Effects	Refs.
Irinotecan (CPT-11)	UGT1A1, UGT1A7, UGT1A9, UGT1A10	Active metabolite, 7-ethyl-10-hydroxycamptothecin (SN-38), undergoes glucuronidation primarily catalyzed by UGTs forming the inactive SN-38 glucuronide.	[55,56,57]
Belinostat	UGT1A1	Primarily catalyzed by UGT1A1, hydroxymethylglucuronidation generates the corresponding glucuronide metabolites, leading to the rapid clearance of the parent drug.	[58,60]
Etoposide	UGT1A1, UGT1A3, UGT1A8	Glucuronidated etoposide is converted into an inactive glucuronide for excretion, diminishing inhibitory effect on topoisomerase II and antitumor efficacy.	[61,62,63]
Epirubicin	UGT2B7	In the liver, epirubicin is extensively metabolized by UGT2B7 into glucuronide conjugates and excreted.	[64,65]
NU/ICRF 505	UGTs	Due to the high hydrophobicity, this compound cannot be directly recognized by ABC transporters and has to undergo UGT-catalyzed glucuronidation to generate a polar metabolite for efflux by MRP1 and MRP3.	[67,68]
Emodin	UGTs	The primary detoxification pathway for emodin relies on UGT-catalyzed glucuronidation coupled with MRP transporters (MRP2 and MRP3/4).	[69,70]
Methotrexate	UGT1A6	Activate the AhR signaling pathway, and then induce the expression of UGT1A6, which catalyzes the glucuronidation inactivation of methotrexate and promotes acquired drug resistance.	[72]
Ribavirin and Cytarabine	UGT1A	In acute myeloid leukemia, GLI1 promotes the glucuronidation modification of chemotherapeutic drugs by enhancing UGT1A protein stability, leading to tumor cell resistance to ribavirin and cytarabine.	[76]

## Data Availability

No new data were created or analyzed in this study. Data sharing is not applicable to this article.
